# Pre-induction cervical assessment using transvaginal ultrasound versus Bishops cervical scoring as predictors of successful induction of labour in term pregnancies: A hospital-based comparative clinical trial

**DOI:** 10.1371/journal.pone.0262387

**Published:** 2022-01-26

**Authors:** Zainab Hananah Abang Abdullah, Kah Teik Chew, V. Ramesh V. Velayudham, Zainab Yahaya, Amilia Afzan Mohd Jamil, Muhammad Azrai Abu, Nur Azurah Abdul Ghani, Nor Azlin Mohamed Ismail

**Affiliations:** 1 Department of Obstetrics & Gynaecology, Universiti Putra Malaysia, Selangor, Malaysia; 2 Department of Obstetrics & Gynaecology, Universiti Kebangsaan Malaysia, Bangi, Malaysia; 3 Department of Obstetrics & Gynaecology, Serdang Hospital, Selangor, Malaysia; University of Illinois Urbana-Champaign, UNITED STATES

## Abstract

**Objective:**

To evaluate the association between transvaginal ultrasound scan of cervix and Bishop’s score in predicting successful induction of labour, cut-off points and patients’ tolerability and acceptance for both procedures.

**Design:**

A comparative clinical trial.

**Setting:**

A tertiary hospital in Selangor, Malaysia.

**Participants:**

294 women planned for elective induction of labour for various indications were included. All women had transvaginal ultrasound to assess the cervical length and digital vaginal examination to assess the Bishop cervical scoring by separate investigators before induction of labour.

**Primary outcome measure:**

To evaluate the association of the cervical length by transvaginal ultrasound scan and Bishop score in predicting successful induction of labour.

**Secondary outcome measure:**

Variables associated with successful induction of labour and patients’ tolerability and acceptance for transvaginal ultrasound scan of cervix.

**Results:**

There was no statistically significant difference among the vaginal and Caesarean delivery groups in terms of mean maternal age, height, weight, body mass index, ethnicity and gestational age at induction. Vaginal delivery occurred in 207 women (70.4%) and 87 women (29.6%) delivered via Caesarean section. There was a high degree of correlation between the cervical length and Bishop score (*r*-value 0.745; p <0.001). Sonographic assessment of cervical length demonstrated a comparable accuracy in comparison to Bishop score. Analysis using ROC curves noted an optimal cut-off value of ≤27mm for cervical length and Bishop score of ≥ 4, with a sensitivity of 69.1% vs 67%, specificity 60.9% vs 55%, and area under the curves (AUCs) of 0.672 and 0.643 respectively (*p* <0.001). Multivariate logistic regression analysis demonstrated that parity (OR 2.70), cervical length (OR 0.925), Bishop score (OR 1.272) and presence of funnelling (OR 3.292) were highly significant as independent predictors of success labour induction. Women also expressed significantly less discomfort with transvaginal ultrasound compared with digital vaginal examination.

**Conclusion:**

Sonographic assessment of cervical measurement predicts the success of induction of labour with similar diagnostic accuracy with conventional Bishop score.

## Introduction

Induction of labour is a fairly routine obstetric procedure worldwide, being performed in approximately 1.4–35% of all deliveries for either maternal and/or fetal reasons [1–3]. Studies comparing induction versus expectant management in post-term pregnancies found that it was associated with a significant reduction in perinatal mortality [4, 5]. Therefore, many studies have looked at various factors which may affect the likelihood of success of labour induction [6]. An important factor is the cervical ripening, whereby certain favourable characteristics of the maternal uterine cervix would readily progress into labour and subsequently result in vaginal birth.

In 1964, Bishop described a cervical scoring system using digital examination to assess cervical ripening [7]. It encompasses several criteria such as the position, consistency, effacement and dilatation of the cervix, and also station of the presenting part, with a maximum score of 13. Studies have shown that a score of more than 8 is favourable for induction of labour, such that it would result in vaginal birth in over 90% of women [3, 8, 9]. However, despite its simplicity and readiness to be performed, there are queries regarding its accuracy due to its subjective nature. Hence scoring may vary according to each clinician. In addition, cervical changes such as funnelling at internal os and cervical length may be difficult to assess in closed cervical os [10]. For these reasons, many sought to find other methods of cervical assessment which may be more objective and reproducible in predicting the success of labour induction.

Cervical evaluation in pregnancies using transvaginal ultrasonography (TVUS) has been documented since 1986, with no additional risks to the mother or the fetus. Multiple studies reported the use of transvaginal ultrasound of cervical length to be a sensitive method for predicting the success of labour induction. Daskalakis et al. reported that cervical length of <27mm measured by transvaginal ultrasound compare to Bishop score were more likely to deliver vaginally with a sensitivity of 76% and specificity of 75.5% [11]. Analysis by Tan et al. also demonstrated that cervical length had a higher sensitivity in predicting failure of induction compared to Bishop score (80% vs 64%), and a slightly higher positive (30% vs. 27%) and negative (89% vs. 83%) predictive values [12]. Furthermore, TVUS was also noted to be better tolerated i.e. lower pain score compared to digital vaginal examination.

However, multiple studies done over the years have shown conflicting results in terms of superiority of TVUS cervix compare to Bishops score [6, 13–15]. Analysis of a study conducted by Chandra et al. failed to demonstrate a significant correlation between cervical measurement by ultrasound and the primary outcome i.e. successful vaginal delivery [13]. Sharma et al. also published a similar study in 2017 and included a comparison of different statistical analyses of previous studies, whereby 9 out the 13 studies showed that cervical length is a better predictor for success of induction of labour, 3 studies demonstrating a comparable significance with Bishop’s scoring, and 1 study found that Bishop’s scoring is superior to cervical length measurement [14]. A recent Cochrane review in 2015 reported that there was no significant difference between TVUS and Bishops score in terms of the main outcomes i.e. vaginal birth or caesarean delivery, and induction to delivery interval [6]. But it is important to note that the evidence was based on trials with small sample size and hence warrants further research to support the use of TVUS for pre-induction cervical evaluation.

Hence, this study was initiated to evaluate the association between TVUS of cervix and Bishop’s Score in predicting successful induction of labour, as well as to determine the optimal cut-off points for cervical length measurement. The secondary endpoint was to assess patients’ tolerability and acceptance for both procedures. This study hypostasised that cervical length measurement by TVUS would be a sensitive tool to predict the outcome of induction of labour, and used in the future to assist in the decision for induction of labour, either by complementing the existing Bishop score or as an alternative to Bishop score as the gold standard pre-induction cervical evaluation.

## Methods

### Participants and recruitment

This was a prospective comparative clinical trial, registered under National Medical Research Register (NMRR) of Malaysia with the Research ID NMRR-18-2118-41815 (IIR) and granted ethical approval by the Medical Research and Ethics Committee (MREC) as well as UKM Human Research Ethics Committee (Project Code FF-2019-368). A total of 294 women were recruited from the Patient Admission Centre (PAC) of Obstetrics & Gynaecology Department, Serdang Hospital in Selangor, Malaysia from January 2019 until January 2020.

Women aged 18 to 40 years old, with term (37 to 42 weeks gestational age) singleton pregnancies, who were planned for elective induction of labour were recruited and followed through the induction process until the delivery of the baby. Those with previous history of uterine surgery, placenta praevia, vaginal bleeding, multiple pregnancies, prelabour rupture of membranes, pre-eclampsia, intrauterine growth restrictions, known allergy towards prostaglandins, intrauterine fetal death, known fetal anomaly and estimated fetal weight >3.8kg by scan were excluded from the study. Written consent was obtained from all recruited women.

### Procedures

**Fig 1:**
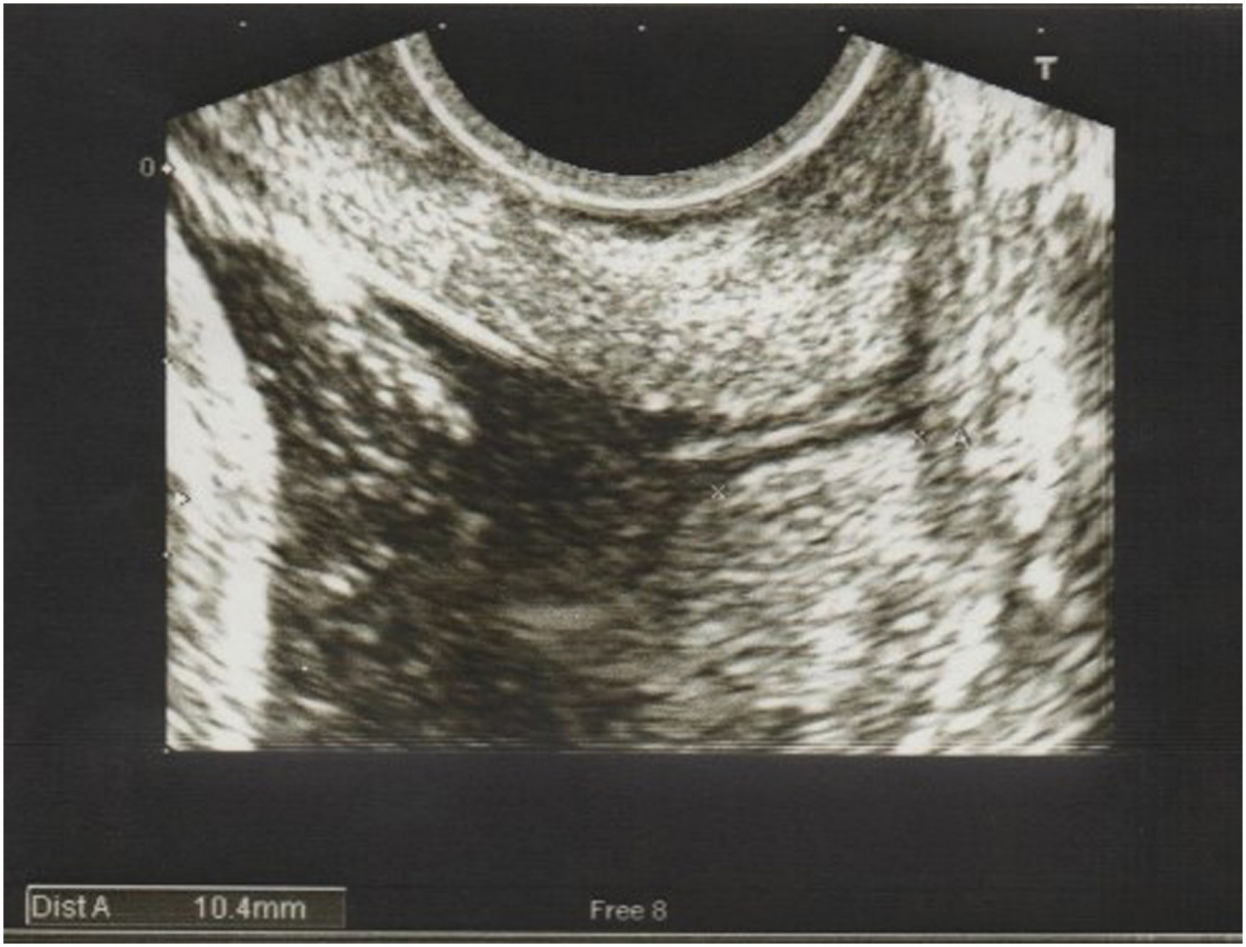
Transvaginal ultrasound scan of cervix, showing length of 10.4 mm.

Upon admission in PAC, a transabdominal ultrasound scan was performed to estimate the fetal weight. The TVUS of cervical length and vaginal examination were performed by two separate investigators (Investigator A and B) and the findings were blinded to each other. A transvaginal ultrasound was done (by Investigator A) with an empty bladder as per Fetal Medicine Foundation guidelines to measure the cervical length (defined as the linear distance between the V-shaped notch at internal os and the triangular area of echodensity at external os, as pictured in (Fig 1). Picture was magnified such that the cervix occupied at least 75% of the image. Three measurements of cervical length were taken over a period of 3 minutes and the best shortest measurement was recorded. The presence of funnelling was also documented, which was a funnel shape appearance at internal cervical os due to internal os dilatation, measuring at least 5mm. The investigator who recruited patients was trained and credentialed in transvaginal ultrasound for cervical length measurement. Subsequently, Bishop’s cervical score was assessed by a different investigator (Investigator B) and each component was documented: os dilatation, cervical length or effacement, station of presenting part, position and consistency of cervix, with a maximum score of 13 (S1 Appendix). Following that, women were asked to score their perception of pain for each procedure (TVUS and vaginal examination) using the 10-point Visual Analogue Scale (VAS), score 0 being ‘no pain’ and score 10 being ‘very painful’. Their sociodemographic and obstetrics data were also collected. Age ≥ 35 years old was defined as advanced maternal age and height ≤ 145cm was classified as short stature.

The patients were subjected for induction of labour by clinicians who were blinded from the transvaginal ultrasound and initial vaginal examination findings, using standard labour induction protocol used at Serdang Hospital either with an intracervical balloon (Foleys) catheter, inflated with 40-60cc of sterile water, placed for a maximum of 24 hours; or Prostaglandin E2 (*Prostin E2*^®^, Dinoprostone 3mg, Pfizer Malaysia) tablets, 2 doses per day at least 6 hours apart, with a maximum of 3 doses in total; or intravenous oxytocin induction, with starting dose of 1–2 mU/min, increased at intervals of 30 min or more, aiming for 4–5 contractions in 10 minutes; or serial induction using a combination of methods above. The choice of induction of labour would be based on clinical risk assessment by the treating clinicians according to the hospital protocol. Augmentation of labour using oxytocin in labour room were done as per hospital protocol. Electronic fetal heart monitoring was performed for all patients.

Primary outcome measured was diagnostic accuracy of the cervical length compared with Bishop score in predicting successful induction of labour. Caesarean delivery was performed for presumed fetal distress based on non-reassuring cardiotocograph tracing; or failure of induction of labour, defined as inability to achieve active phase of labour (cervical dilatation of >4cm) after 24 h of prostaglandin administration ± 12 h of oxytocin infusion; or poor progress, which is defined as progress in cervical dilatation by less than 2 cm after 4 hours of oxytocin.

### Statistical analysis

Data was analysed using statistical software SPSS (Version 25.0. Armonk, NY: IBM Corp.). For descriptive analysis, the means, standard deviation, median and interquartile range (IQR) were calculated. Shapiro-Wilk test was used to evaluate the normality of the data variables. Inferential analysis was done using Independent Samples *t*-test for continuous parametric variables, Mann-Whitney-U test for non-parametric variables and Fisher’s exact test for categorical data. An analysis via Receiver operator Characteristics (ROC) curve was performed to evaluate the optimal threshold value for cervical length measurement and Bishop score in predicting success of induction of labour i.e. vaginal delivery. The area under the curve (AUC) with the respective confidence intervals (CI) were obtained. The diagnostic characteristics of these threshold values were assessed using sensitivity, specificity, positive and negative predictive values, positive and negative likelihood ratio, with 95% confidence intervals, to identify the ability to accurately predict vaginal delivery. A p-value of <0.05 was considered as statistically significant. Univariate and multivariate logistic regression analysis were performed to determine the relationship between successful induction of labour with various variables.

### Patient and public involvement

Patients or the public were not involved in the design, or conduct, or reporting, or dissemination plans of this study.

## Results

**Fig 2:**
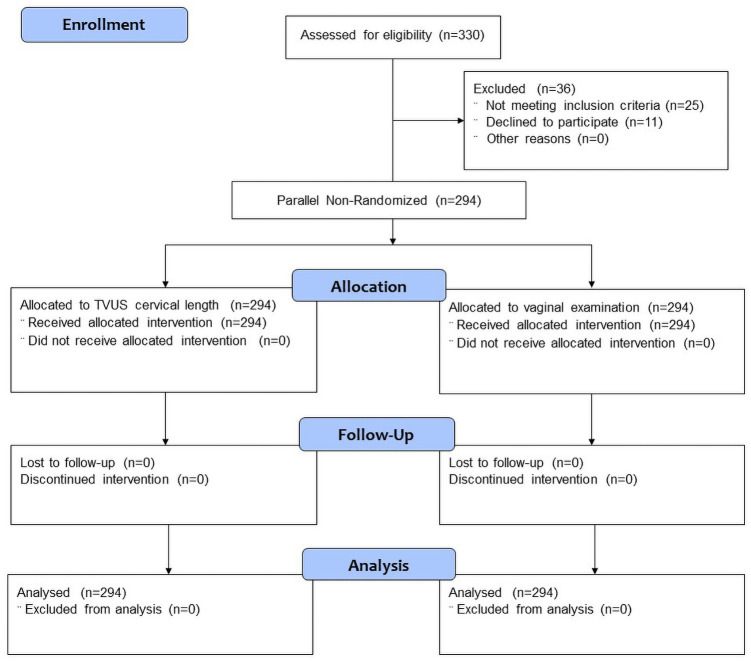
Flow chart of participants.

A total of 330 women were assessed for eligibility, 11 women declined to participate and 25 women were excluded as not meeting the inclusion criteria (Fig 2). Eventually, a total of 294 women were recruited and analysed, which included 132 nulliparous (44.9%) and 162 multiparous (55.1%) women. Half of them were induced for diabetes (49.3%), followed by post-dates pregnancies, defined as ≥ 41 completed weeks (12.6%), small for gestational age, SGA (11.6%), oligohydramnios (10.2%), hypertension (6.8%), reduced fetal movement (6.5%) and other reasons, such as subfertility and late confirmation of pregnancy beyond second trimester (3.1%). Maternal demographic characteristics were shown in Table 1. There was no statistically significant difference among the vaginal and Caesarean delivery groups in terms of mean maternal age (30.28 ± 5.17 vs 30.43 ± 4.62 years, *p* = 0.821), mean height (156.38 ± 5.69 vs 156.18 ± 5.34 cm, *p* = 0.787), mean weight (63.56 ± 13.97 vs 68.54 ± 18.07 kg, *p* = 0.11), body mass index (25.90 ± 5.25 vs 28.05 ± 6.99 kg/m^2^, *p* = 0.11), ethnicity (*p* = 0.055), mean gestational age at induction (39.18 ± 1.18 vs 39.06 ± 1.23 weeks, *p* = 0.429), estimated fetal weight by transabdominal scan (3021.23 ± 284.75 vs 3068.94 ± 261.66 gm, *p* = 0.18), as well as the indications of induction of labour (*p* = 0.533).

**Table 1:** Maternal demographic characteristics between vaginal and Caesarean deliveries.

Variables	Vaginal Delivery *n* = 207	Caesarean Section *n* = 87	*p*-value
Age, in years (Mean, SD*)	30.28 ± 5.17	30.43 ± 4.62	0.821
< 35 years old (*n*)	159 (76.8%)	66 (75.9%)	0.881
≥ 35 years old (*n*)	48 (23.2%)	21 (24.1%)	
Height (cm) (Mean, SD)	156.38 ± 5.69	156.18 ± 5.34	0.787
≤ 145 cm (*n*)	6 (2.9%)	2 (2.3%)	1.0
>145 cm (*n*)	201 (97.1%)	85 (97.7%)	
Weight (kg) (Mean, SD)	63.56 ± 13.97	68.54 ± 18.07	0.11
BMI (kg/m^2^) (Mean, SD)	25.90 ± 5.25	28.05 ± 6.99	0.11
Parity (Median, IQR**)	1 (2)	0 (1)	<0.001
Nulliparous (*n*)	78 (37.7%)	54 (62.1%)	<0.001
Parous, ≥1 (*n*)	129 (62.3%)	33 (37.9%)	
Ethnicity (*n*)			
Malay	165 (79.7%)	68 (78.2%)	0.055
Indian	11 (5.3%)	9 (10.3%)	
Chinese	15 (7.3%)	9 (10.3%)	
Others	16 (7.7%)	1 (1.2%)	
Gestational age (weeks)	39.18 ± 1.18	39.06 ± 1.23	0.429
Estimated fetal weight (gm)	3021.23 ± 284.75	3068.94 ± 261.66	0.18
Indications (*n*)			
Diabetes	105 (50.7%)	40 (46.0%)	0.533
Post dates	27 (13.0%)	10 (11.5%)	
Small for gestational age	27 (13.0%)	7 (8.0%)	
Oligohydramnios	18 (8.7%)	12 (13.8%)	
Hypertension	12 (5.8%)	8 (9.2%)	
Reduced fetal movement	12 (5.8%)	7 (8.0%)	
Others*	6 (3.0%)	3 (3.5%)	

Analysis was by Independent *t*-test for continuous parametric variables, Mann-Whitney-U test for non-parametric variables, Fisher’s exact test for categorical data.

*Standard deviation (SD).

**Interquartile range (IQR).

Successful induction of labour i.e. vaginal delivery occurred in 207 women (70.4%). A total of 87 women (29.6%) delivered via Caesarean Section, with the indication of fetal distress (38 women, 12.9%), poor progress of labour (32 women, 10.9%) and failed induction of labour (17 women, 5.8%). There was a high degree of correlation between the cervical length and Bishop score, with *r*-value of 0.745, *p* <0.001. Variables such as parity, cervical length, presence of funnelling and Bishop score were associated with successful induction of labour. Parous women, who were defined as women with one or more previous vaginal deliveries were significantly associated with successful vaginal delivery (*p* = 0.001). Mean cervical length for those delivered vaginally were significantly shorter by 4 mm as compared to those delivered via Caesarean (*p* = 0.001). Similarly, the presence of funnelling correlated well with successful vaginal delivery (*p* = 0.001). In the vaginal delivery group, the median Bishop score (5, IQR 3) was significantly different from the median value in the Caesarean group (3, IQR 3) (*p* = 0.001). Women who delivered vaginally also had significantly shorter induction to delivery interval (30.33 ± 27.82 hours vs 43.53 ± 36.00 hours, p = 0.003), as well as smaller babies (2971.55 ± 394.67 gm vs 3123.28 ± 409.94 gm, p = 0.003), compared with women who underwent Caesarean. Analysis comparing the method of induction either single or combination induction agents between women who delivered vaginally and via Caesarean found no significant different with *p*-value of 1.0 (Table 2).

**Table 2:** Induction & delivery parameters between groups.

Variables	Vaginal Delivery *n* = 207	Caesarean Section *n* = 87	*p*-value
Cervical length (mm)	23.96 ± 7.73	28.36 ± 7.18	0.001
(Mean, SD)			
Cervical funnelling			
Present (*n*, %)	136 (65.7%)	32 (36.8%)	0.001
Absent (*n*, %)	71 (34.3%)	55 (63.2%)	0.001
Bishop score (Median, IQR)	5 (3)	3 (3)	0.001
Method of induction			
Foleys (*n*)	14	6	1.0
PGE2 (*n*)	89	25	0.026
Oxytocin (*n*)	54	13	0.047
Single agent (*n*, %)	156 (75.4%)	45 (51.7%)	1.0
Combination (*n*, %)	51 (24.6%)	42 (48.3%)	1.0
Induction to delivery	30.33 ± 27.82	43.53 ± 36.00	0.003
interval (hours)			
Birthweight (gm)	2971.55 ± 394.67	3123.28 ± 409.94	0.003

Analysis was by Independent *t*-test for continuous parametric variables, Mann-Whitney-U test for non-parametric variables, Fisher’s exact test for categorical data.

**Fig 3:**
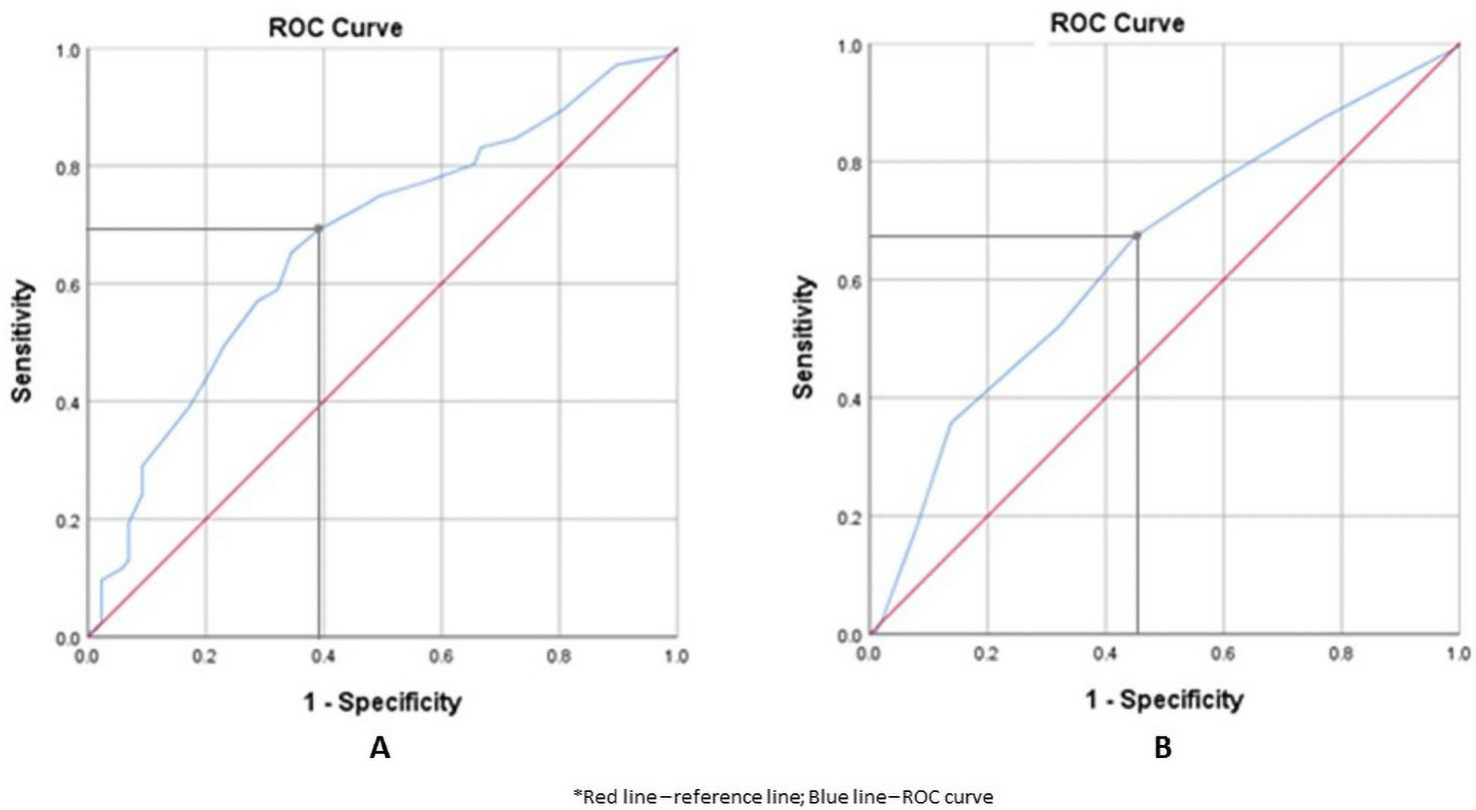
Receiver Operating Characteristics (ROC) curves for cervical length (A) and Bishop score (B).

The ROC curves were constructed to determine the optimal cut-off value of cervical length and Bishop score to predict a successful induction of labour (Fig 3). There was a significant relationship between these variables and prediction of vaginal delivery as both curves were above the 45° line. The curve for cervical length showed an optimal cut-off value of 27 mm corresponding to a sensitivity of 69.1% (95% CI 62.2–75.2) and specificity of 60.9% (95% CI 49.8–71.0), whereas the optimal cut-off value for Bishop score was 4, with a sensitivity of 67% (95% CI 60.2–73.4) and specificity of 55% (95% CI 44.2–65.7) (Table 3). The area under the curve (AUC) for cervical length and Bishop score were similar at 0.672 (95% CI 0.606–0.7390) and 0.643 (95% CI 0.575–0.710) respectively and both were highly significant with a *p*-value of <0.001.

**Table 3:** ROC corresponding AUC, sensitivity, specificity and significance.

		Sensitivity (%)	Specificity (%)	Area under curve, AUC (95% CI)	Standard Error	*p*-value
Cervical length (mm)	≤ 20	34.8	86.2	0.672 (0.606–0.739)	0.034	<0.001
	≤ 21	39.1	82.8			
	≤ 22	44.9	79.3			
	≤ 23	49.3	77.0			
	≤ 24	57.0	71.3			
	≤ 25	58.9	67.8			
	≤ 26	65.2	65.5			
	**≤ 27**	**69.1**	**60.9**			
	≤ 28	73.4	52.9			
	≤ 29	74.9	50.6			
	≤ 30	77.3	42.5			
Bishop Score	≥ 2	87.4	23.0	0.643 (0.575–0.710)	0.035	<0.001
	≥ 3	76.3	41.4			
	**≥ 4**	**67.1**	**55.2**			
	≥ 5	52.2	67.8			
	≥ 6	35.7	86.2			
	≥ 7	18.4	92.0			
	≥ 8	2.9	97.7			

The diagnostic characteristics of cervical length at the specific cut-off value ≤ 27 mm were similar to Bishop score ≥ 4 in predicting successful induction of labour, with a *p*-value of <0.001. The sensitivity was 69% for cervical length vs 67% for Bishop score, specificity 60.9% vs 55.2%, positive likelihood ratio (+LR) 1.77 vs 1.50, and negative likelihood ratio (–LR) 0.51 vs 0.60 respectively (Table 3).

Multivariate logistic regression analysis was performed to evaluate the relationship between various variables and successful induction of labour (Table 4). The degree of the association was determined by *p*-value for the odds ratio (OR). OR > 1 demonstrates a positive association, such that an increase in value would result in higher success of induction of labour, whereas an OR < 1, indicates a negative correlation, meaning an increase in value would result in lower success of induction of labour. Both cervical length and Bishop score had significant correlation for predictor of successful induction, with an OR of 0.925 (95% CI 0.892–0.959) and 1.272 (95% CI 1.121–1.443) respectively, with *p* <0.001. Other significant factors for predicting vaginal delivery included parity, presence of funnelling, maternal weight and BMI (*p* <0.001).

**Table 4:** Relationship between various variables and successful induction of labour i.e. vaginal delivery.

Variable	Odds Ratio (OR)	95% Confidence interval	*p*-value
Age	0.994	0.946–1.045	0.820
Height	1.006	0.962–1.052	0.786
Weight	0.980	0.964–0.996	0.013
BMI	0.941	0.902–0.982	0.005
Parity	2.706	1.615–4.535	< 0.001
Cervical length	0.925	0.892–0.959	< 0.001
Bishop score	1.272	1.121–1.443	< 0.001
Presence of funnelling	3.292	1.954–5.547	< 0.001

Using the visual analogue scale (VAS), women had better tolerability with transvaginal ultrasound scan by expressing significantly less pain or discomfort (median score 2, IQR 3), compared with assessment using digital vaginal examination (median score 5, IQR 4), with a *p*-value of <0.001.

## Discussion

The assessment of cervical status prior to induction is important in order to determine the chances of successful vaginal delivery. Having this knowledge could also guide clinicians to carefully select and counsel women in order to optimise the outcome of labour induction. This study demonstrated that pre-induction transvaginal cervical length measurement was a significant independent predictor of successful induction of labour (p<0.001). This was in agreement with previous studies which looked at the role of pre-induction cervical length assessment [11, 14, 16–20]. In addition, our analysis showed a high degree of correlation between cervical length and Bishop score with *r*-value of 0.745 (*p* <0.001), hence providing evidence for the potential use of sonographic assessment of cervix as a method to predict successful induction. This was supported by Eggebo et al. who also reported positive correlation between ultrasound measurements and elements of Bishop score [9].

Based on the analysis obtained from the ROC curves, a threshold value of ≤ 27 mm for cervical length and Bishop score ≥ 4 were associated with more successful induction (*p* <0.001). The diagnostic characteristics for both methods were comparable, with sensitivity of 69% for cervical length vs 67% for Bishop score, specificity 60.9% vs 55.2%, positive LR and negative LR. In addition, the areas under the curves (AUCs) were also similar at 0.672 and 0.643 respectively. This corresponded to data published by Alanwar et al., who conducted a similar study on 320 women, and found that both cervical length measurement and Bishop score had similar predictive value for outcome of induction [21]. They reported the best cut-off measurement was 23 mm for cervical length and 5 for Bishop score, with similar AUCs of 0.694 and 0.623 respectively [21].

Multivariate logistic regression analysis of our data demonstrated an Odds Ratio of 0.92 (0.89–0.95) for cervical length and 1.27 (1.12–1.44) for Bishop score in predicting successful induction. This indicated that an increment of 1 mm in cervical length was associated with 7.5% decrease in odds of having vaginal delivery. Likewise, an increment of one score for Bishops score would increase the odds of having vaginal delivery by 27%. Both methods were statistically significant with *p*-value of <0.001.

Whilst our study demonstrated a comparable result for both methods, previous studies looking at the role of sonographic assessment of cervical length versus Bishop Score had reported conflicting results. Pandis et al. looked at 240 women and found that both cervical length of less than 28mm and Bishop score more than 3 were independent predictors of vaginal delivery within 24 hours of induction [17]. However further analysis using Kaplan-Meier survival curves demonstrated that cervical length was a better predictor compared to Bishop score. Similar results were published by Maitra et al. and Sharma et al., both quoting a cervical length cut-off of less than 30mm, as a better predictor of successful induction compared with Bishop score [10, 14]. A Cochrane review in 2015 reported no clear difference between cervical length and Bishop score in predicting outcome of labour induction [6]. However, these were moderate quality evidence from two small trials involving a total of 234 women only, therefore providing insufficient evidence to support the use of sonographic assessment of cervix over Bishop score. In contrast, Groeneveld et al. examined 110 women and found that Bishop score of 3 or more was an independent predictor of vaginal delivery, whereas cervical length was not a predictor of success in either nulliparous or multiparous women [22]. However, their study had multiple indications of induction with small number of cases each, which may had affected the analysis. Inter-observer variation was the contributing factor for the conflicting results from these studies.

One of the cervical changes with regards to the onset of labour is effacement, which refers to the shortening of the cervical canal [23]. Previous sonographic researches utilising MRI and ultrasound found that cervical effacement begins at the internal os and proceeds downwards, subsequently allowing protrusion of fetal membrane into upper cervical canal i.e. funnelling. Changes in the composition and microstructure of the cervix lead to softening and funneling of the cervix, therefore allowing progression of labour to occur more readily. Multivariate logistic regression analysis of our data found that the presence of funnelling was a significant predictor of successful induction, with an odds ratio (OR) of 3.2 (*p* <0.001). This showed that women with presence of funnelling on ultrasound had 3.2 times increased chance of having a vaginal delivery. Although this finding concurred with the analysis by Chung et al., who reported that funnelling was significantly associated with successful vaginal delivery after adjustment for cervical length and Bishop score (OR 2.70, 95% CI 1.02–7.10; p = 0.04), many previous studies had demonstrated otherwise [24]. Multiple logistic regression analysis by Keepanasseril et al. demonstrated lack of association between funnelling and successful vaginal birth (OR 1.018, 95% CI 0.975–1.063; *p* = 0.415) [18]. Kant et al. found that percentage of funnelling was statistically insignificant in predicting outcome of labour induction (*p* = 0.222) [25]. On the other hand, Bajpai et al. incorporated funnelling length and width into their Manipal Cervical Scoring System using transvaginal ultrasound to achieve an excellent predictive value in predicting the outcome of labour induction with an area under the ROC curve (AUC) of 0.940 (95% CI 0.876–0.977; *p* <0.0001) [26]. Funnelling alone was found to be insignificant as a predictor. All these studies shared a similar confounding bias which was inter-observer bias. However, a latest study with no inter-observer variation revealed that presence of cervical funneling was similar as efficient as Bishop score and cervical length, as an independent predictor for successful induction of labour [27]. Another significant predictor is parity, whereby our study showed that one previous vaginal delivery will increase the odds of having a successful induction by 2.7 times. This was also an established independent factor from other published studies [12, 26, 28, 29].

Over the years, Bishop scores remained the standard method of cervical assessment as it is inexpensive, readily available and is a simple method which does not require any special setting or equipment. In clinical practice however, it can be highly subjective depending on the experience of the operator with high inter and intra observer variability. This inevitably affects the sensitivity, specificity as well as predictive values of this method to predict the outcome of labour. This study portrayed that transvaginal ultrasound of cervix has equivalent diagnostic characteristics as the conventional Bishop score. Therefore, in a centre where ultrasound facilities are available, clinicians could opt to practice this method as an alternative or in addition to Bishop score in assessing women prior to induction.

Additional strengths advocating the role of sonographic measurement of the cervix include allowing a more objective, accurate cervical assessment and it is reproducible. Images can be printed or saved digitally for various purposes such as for reference, as part of medicolegal documentation as well as a visual aid to improve patient counselling. Furthermore, these images can be used as a learning tool for training healthcare workers who may not have much experience with performing the Bishop cervical scoring such as medical students, sonographers, midwives and others. Moreover, transvaginal ultrasound has the additional advantage of visualising the whole length of the cervix, while assessing the internal os for presence of funnelling, which would be difficult with a digital vaginal examination.

Another key point to highlight is that women seemed to tolerate transvaginal ultrasound scan better compared with digital vaginal examination by expressing significantly less discomfort (Median score 2, IQR 3 vs median score 5, IQR 4) (*p* <0.001). This validated the previous study by Tan et al. [12]. Therefore, it was inferred that women would be more accepting of this method if it were to be used as an alternative for cervical assessment. This is an invaluable tool particularly for women who are unable to tolerate pain with vaginal examination. Gunes et al. revealed a positive association between discomfort during vaginal examination and emotional violence as well as post-traumatic stress disorder [30]. For this reason, utilising transvaginal ultrasound for cervical assessment instead of vaginal examination for Bishop score may be the better option. Reducing pain and allaying women’s fear of vaginal examination would in turn improve patients’ compliance as well as optimise the outcome of pregnancy.

Despite various evidence demonstrating the potential benefit of transvaginal ultrasound assessment of cervix in predicting successful labour induction, there are some limitations to this method. First and foremost, an ultrasound scan machine, specifically with the transvaginal ultrasound probe is needed, which may not be available at all centres due to the high cost. Additionally, transvaginal ultrasound requires appropriate training and credentialing. This is due to the need for proper measurement technique which is more difficult at term, particularly with fetal head engagement and therefore the alignment of the cervix is distorted [17]. This may be the reason some clinicians continue to practice Bishop Score at their respective centres as the standard cervical assessment prior to induction of labour.

## Strengths and limitations of the study

The strengths of this study include the consistent follow up of all women until the delivery of the babies, with no withdrawals from study or loss to follow up, therefore minimising attrition bias. Inter-observer variability was also eliminated as the TVUS was performed by the same investigator. In addition, the clinicians managing the induction and delivery process were blinded to the initial assessment by the investigators. There were some limitations that were identified for this study. Firstly, this study involved only a sample of population from a single medical centre and may not depict the rest of the population. Furthermore, the sample size calculated was not for the comparison between these two models. In addition, we did not specify the induction methods as per other studies. Different induction methods may have an effect on the duration and outcome of labour. We also did not evaluate other sonographic parameters of cervix such as presence of wedging, posterior cervical angle or distance of presenting part to external os, which may have additional value in predicting successful induction of labour. Further study in the future is needed to appraise the use of transvaginal ultrasound of cervix involving a larger sample.

## Conclusion

In conclusion, in a setting where transvaginal ultrasound scan is available, utilising this method to evaluate the likelihood of successful induction of labour in term pregnancies is the alternative to the current Bishop’s cervical scoring. This study demonstrated that cervical length was a highly significant independent predictor of successful induction. An optimal cut-off value of ≤ 27 mm had comparable diagnostic characteristics with Bishop score ≥ 4 to predict vaginal delivery. Nevertheless, the result of this study need to be interpreted in caution as this was a single centre study and different induction methods may have an effect on the duration and outcome of labour. Women had better tolerability with sonographic assessment of cervix using transvaginal ultrasound scan as evidenced by significantly less pain score compared with digital vaginal examination.

## Supporting information

S1 Appendix The study protocol. (DOCX)

S1 Checklist TREND statement checklist. (DOC)

## References

[1] Getahun D. Epidemiologic considerations: scope of problem and disparity concerns. Clinical obstetrics and gynecology. 2014;57(2):326. doi: 10.1097/GRF.0000000000000021 24614814 PMC4008709

[2] Vogel JP, Souza JP, Gülmezoglu AM. Patterns and outcomes of induction of labour in Africa and Asia: a secondary analysis of the WHO Global Survey on Maternal and Neonatal Health. PloS one. 2013;8(6):e65612. doi: 10.1371/journal.pone.0065612 23755259 PMC3670838

[3] Marconi AM. Recent advances in the induction of labor. F1000Research. 2019;8. doi: 10.12688/f1000research.17587.1 31723412 PMC6823899

[4] Cole R, Howie P, Macnaughton M. Elective induction of labour: a randomised prospective trial. The Lancet. 1975;305(7910):767–70. doi: 10.1016/s0140-6736(75)92435-6 48000

[5] Sue-A-Quan AK, Hannah ME, Cohen MM, Foster GA, Liston RM. Effect of labour induction on rates of stillbirth and cesarean section in post-term pregnancies. Cmaj. 1999;160(8):1145–9. 10234344 PMC1230266

[6] Ezebialu IU, Eke AC, Eleje GU, Nwachukwu CE. Methods for assessing pre‐induction cervical ripening. Cochrane Database of Systematic Reviews. 2015(6). doi: 10.1002/14651858.CD010762.pub2 26068943 PMC4473357

[7] Bishop EH. Pelvic scoring for elective induction. Obstet Gynecol. 1964;24(2):266–8. 14199536

[8] Baacke KA, Edwards RK. Preinduction cervical assessment. Clinical obstetrics and gynecology. 2006;49(3):564–72. doi: 10.1097/00003081-200609000-00016 16885663

[9] Eggebø TM, Økland I, Heien C, Gjessing LK, Romundstad P, Salvesen KÅ. Can ultrasound measurements replace digitally assessed elements of the Bishop score? Acta obstetricia et gynecologica Scandinavica. 2009;88(3):325–31. doi: 10.1080/00016340902730417 19172418

[10] Maitra N, Sharma D, Agarwal S. Transvaginal measurement of cervical length in the prediction of successful induction of labour. Journal of Obstetrics and Gynaecology. 2009;29(5):388–91. doi: 10.1080/01443610802712900 19603314

[11] Daskalakis G, Thomakos N, Hatziioannou L, Mesogitis S, Papantoniou N, Antsaklis A. Sonographic cervical length measurement before labor induction in term nulliparous women. Fetal diagnosis and therapy. 2006;21(1):34–8. doi: 10.1159/000089045 16354972

[12] Tan P, Vallikkannu N, Suguna S, Quek K, Hassan J. Transvaginal sonographic measurement of cervical length vs. Bishop score in labor induction at term: tolerability and prediction of Cesarean delivery. Ultrasound in Obstetrics and Gynecology: The Official Journal of the International Society of Ultrasound in Obstetrics and Gynecology. 2007;29(5):568–73. doi: 10.1002/uog.4018 17444553

[13] Chandra S, Crane JM, Hutchens D, Young DC. Transvaginal ultrasound and digital examination in predicting successful labor induction. Obstetrics & Gynecology. 2001;98(1):2–6. doi: 10.1016/s0029-7844(01)01386-2 11430948

[14] Sharma SK, Nagpal M, Thukral C. Evaluation of pre induction scoring by clinical examination vs transvaginal sonography. International Journal of Reproduction, Contraception, Obstetrics and Gynecology. 2017;6(1):229.

[15] Gabriel R, Darnaud T, Gonzalez N, Leymarie F, Quereux C. Transvaginal ultrasonography of the uterine cervix before induction of labor. Gynecologie, obstetrique & fertilite. 2001;29(12):919–23. 11802557 10.1016/s1297-9589(01)00244-2

[16] Tan PC, Suguna S, Vallikkannu N, Hassan J. Ultrasound and clinical predictors for Caesarean delivery after labour induction at term. Australian and New Zealand journal of obstetrics and gynaecology. 2006;46(6):505–9. doi: 10.1111/j.1479-828X.2006.00650.x 17116055

[17] Pandis G, Papageorghiou A, Ramanathan V, Thompson M, Nicolaides K. Preinduction sonographic measurement of cervical length in the prediction of successful induction of labor. Ultrasound in Obstetrics and Gynecology: The Official Journal of the International Society of Ultrasound in Obstetrics and Gynecology. 2001;18(6):623–8. doi: 10.1046/j.0960-7692.2001.00580.x 11844202

[18] Keepanasseril A, Suri V, Bagga R, Aggarwal N. Pre‐induction sonographic assessment of the cervix in the prediction of successful induction of labour in nulliparous women. Australian and New Zealand journal of obstetrics and gynaecology. 2007;47(5):389–93. doi: 10.1111/j.1479-828X.2007.00762.x 17877596

[19] Panchampreet K, Manpreet K, Manjula R, Mini M. Role of transvaginal sonography in preiduction cervical assessment. Is it helpful? International Journal of Contemporary Medical Research. 2017;4(7):1549–54.

[20] Said M, Khalil O, Mansy A, Farag M. Sonographic cervical canal length and/or a bishop score assessment as a predictor for successful induction of labor. The Egyptian Journal of Fertility of Sterility. 2018;22(2):38–43.

[21] Alanwar A, Hussein SH, Allam HA, Hussein AM, Abdelazim IA, Abbas AM, et al. Transvaginal sonographic measurement of cervical length versus Bishop score in labor induction at term for prediction of caesarean delivery. The Journal of Maternal-Fetal & Neonatal Medicine. 2019:1–8. doi: 10.1080/14767058.2019.1659770 31438737

[22] Groeneveld Y, Bohnen A, Van Heusden A. Cervical length measured by transvaginal ultrasonography versus Bishop score to predict successful labour induction in term pregnancies. Facts, views & vision in ObGyn. 2010;2(3):187. 25013711 PMC4090590

[23] Nott JP, Bonney EA, Pickering JD, Simpson NA. The structure and function of the cervix during pregnancy. Translational Research in Anatomy. 2016;2:1–7.

[24] Chung SH, Kong MK, Kim EH, Han SW. Sonographically accessed funneling of the uterine cervix as a predictor of successful labor induction. Obstetrics & gynecology science. 2015;58(3):188–95. 26023667 10.5468/ogs.2015.58.3.188 PMC4444514

[25] Kant RH, Bashir A, Gupta S. Study of Transvaginal Sonographic Assessment of Cervix in Predicting the Success of Labour Induction in Nulliparous Women. JK Science. 2016;18(1).

[26] Bajpai N, Bhakta R, Kumar P, Rai L, Hebbar S. Manipal cervical scoring system by transvaginal ultrasound in predicting successful labour induction. Journal of clinical and diagnostic research: JCDR. 2015;9(5):QC04. doi: 10.7860/JCDR/2015/12315.5970 26155521 PMC4484113

[27] Kim YN, Kwon JY, Kim EH. Predicting labor induction success by cervical funneling in uncomplicated pregnancies. Journal of Obstetrics and Gynaecology Research. 2020;46(7):1077–83. doi: 10.1111/jog.14270 32390283 PMC7384017

[28] Rane S, Guirgis R, Higgins B, Nicolaides K. Models for the prediction of successful induction of labor based on pre-induction sonographic measurement of cervical length. The Journal of Maternal-Fetal & Neonatal Medicine. 2005;17(5):315–22. doi: 10.1080/14767050500127690 16147844

[29] Taha OT, Elprince M, Atwa KA, Elgedawy AM, Ahmed AA, Khamees RE. Antenatal cervical length measurement as a predictor of successful vaginal birth. BMC Pregnancy and Childbirth. 2020;20(1):1–6. doi: 10.1186/s12884-020-02878-z 32228499 PMC7106757

[30] Güneş G, Karaçam Z. The feeling of discomfort during vaginal examination, history of abuse and sexual abuse and post‐traumatic stress disorder in women. Journal of clinical nursing. 2017;26(15–16):2362–71. doi: 10.1111/jocn.13574 27603931

