# Supplementary material for: Pre-induction cervical assessment using transvaginal ultrasound versus Bishops cervical scoring as predictors of successful induction of labour in term pregnancies: A hospital-based comparative clinical trial
Source: PLoS One. 2022 Jan 26;17(1):e0262387. doi: 10.1371/journal.pone.0262387 (PMC8791481; doi:10.1371/journal.pone.0262387)
Supplement: S1 Appendix — (DOCX) [file pone.0262387.s001.docx]

**Study Protocol**

1. Patient will be admitted from PAC to ward for the purpose on induction of labour, between Monday to Friday.
2. Patient will be recruited according to the inclusion and exclusion criteria.
3. Patient will be approached and explained regarding the study, and consent form signed if the patient is agreeable.
4. The patient will then have a transabdominal ultrasound scan to estimate the fetal weight.
5. Transvaginal ultrasound is done to measure the cervical length (by Investigator A), as per Fetal Medicine Foundation guidelines:
   1. The woman empties her bladder and is then placed in the dorsal lithotomy position.
   2. The ultrasound probe is introduced in the vagina and directed in the anterior fornix. Care is taken to avoid exerting undue pressure on the cervix, which may artificially increase the length.
   3. A sagittal view of the cervix is obtained and the endocervical mucosa (which may be of increased or reduced echogenicity compared to the cervix) is used as a guide to the true position of the internal os, thereby avoiding confusion with the lower segment of the uterus.
   4. The calipers are used to measure the linear distance between the triangular area of echodensity at the external os and the V-shaped notch at the internal os.
   5. Each examination should be performed over a period of 2-3 minutes. In about 1% of cases the cervical length may change due to uterine contractions and in such cases the shortest measurement should be recorded.
   6. Presence of funnelling is also noted and measured. Funnelling is defined as funnel shape appearance at internal cervical os due to internal os dilatation, measuring at least 5mm (9).
   7. Ultrasound will be done by allocated medical staff with at least 1 year of ultrasound scan experience.
6. Vaginal examination for cervical assessment using Bishop’s score is done after the ultrasound (by Investigator B).
   1. The women lies in the dorsal lithotomy position.
   2. After hand washing, the staff will wear sterile gloves and the perineum is cleaned using at least 3 wet cotton swabs.
   3. The index and middle fingers of the provider’s dominant hand is inserted gently into the vagina, in a downward and backward direction along the anterior vaginal wall to locate the cervix (11).
   4. At the cervical opening, the two fingers are spread apart to measure the dilatation of the cervix and documented in centimetres (cm).
   5. Effacement is assessed by the length of cervix and degree to which it protrudes into the vagina.
   6. The position of the cervix is described as posterior, axial or anterior.
   7. The cervical consistency is assessed and described as firm, medium, soft.
   8. Station is the distance between the presenting part of the fetus and the patient’s ischial spines in centimetres (cm). Presenting part above the spines will be (-2cm, -1cm) and below the spines referred to as (+1cm, +2cm), or at the level of ischial spines i.e. station 0.
7. After both procedures, the patient will be asked about her perception and tolerability of both methods using a 10 point Visual Analogue Scale, 0 being no pain and 10 being very painful.
   1. The pain Visual Analogue Scale (VAS) is a generic, commonly used unidimensional pain questionnaire to measure pain intensity (12). It is a continuous scale comprised of a horizontal line, usually 10 centimeters in length, anchored by 2 descriptors of each symptom extreme.
   2. Score of 0 is described as “no pain” followed by the other end of the horizontal line, tagged by a maximum score of 10, described as” “worst imaginable pain”.
8. Patient will then subjected for induction of labour using standard hospital protocol, either using intracervical balloon (Foleys) catheter, or using prostaglandin E2 (Prostin) tablets, with a maximum of 2 doses per day, or oxytocin induction.
9. All data are documented using the data collection form, each labelled with a specific study number. Names OR Identification Card numbers will not be documented in the data collection forms to protect confidentiality.
   1. These documents will be stored in the office of the Fetomaternal Unit, Hospital Serdang and can only be accessed by the principal investigators and the research team.
